# A Non-Intrusive GMA Welding Process Quality Monitoring System Using Acoustic Sensing

**DOI:** 10.3390/s90907150

**Published:** 2009-09-09

**Authors:** Eber Huanca Cayo, Sadek Crisostomo Absi Alfaro

**Affiliations:** Automation and Control Group in Manufacturing Processes – GRACO, University of Brasilia, Faculty of Technology, Department of Mechanical / Mechatronic Engineering, Campus Universitario Darcy Ribeiro–Asa Norte 70910-900-Brasilia/DF-Brazil; E-Mail: sadek@unb.br

**Keywords:** non-intrusive sensing, acoustic welding monitoring, quality control

## Abstract

Most of the inspection methods used for detection and localization of welding disturbances are based on the evaluation of some direct measurements of welding parameters. This direct measurement requires an insertion of sensors during the welding process which could somehow alter the behavior of the metallic transference. An inspection method that evaluates the GMA welding process evolution using a non-intrusive process sensing would allow not only the identification of disturbances during welding runs and thus reduce inspection time, but would also reduce the interference on the process caused by the direct sensing. In this paper a nonintrusive method for weld disturbance detection and localization for weld quality evaluation is demonstrated. The system is based on the acoustic sensing of the welding electrical arc. During repetitive tests in welds without disturbances, the stability acoustic parameters were calculated and used as comparison references for the detection and location of disturbances during the weld runs.

## 1. Introduction

Welding is the most used manufacturing process in the metallic construction industry. Gas metal arc welding—GMAW, also known as MIG/MAG, is the most used welding process due to its diverse advantages, such as the high rate of metallic transference, elevated penetration and facility to weld in all positions. When the use of this welding process grew on industrial scales, the exigencies and requirements of weld quality monitoring also multiplied. By monitoring arc voltage and welding current allowing the detection of the arc perturbations during the welding process and depending of its profile, these perturbations can be interpreted as defects on the welded joints. On-line detection and localization of the defects reduces their severity and the time requirements of the quality control tests. Most commercial equipment for arc voltage and welding current monitoring use sensors based in voltage divisors and the Hall effect and they are installed directly on the welding process. The sensors connected directly on the welding process present two considerable disadvantages: the stability of the welding process, due to its high sensibility, can be interfered with by sensors with electrical connections that alter the electrical arc impedance and generate undesirable arc instabilities and the electromagnetic arc interferences alter considerably the measurements made by the sensors. The electrical arc generates physical phenomena like luminosity, infrared radiation electromagnetic fields and sound pressure. It is known that the specialized welders use an acoustic and visual combination for the monitoring and control of the welding process [1]. Over the last 70 years’ measurement of electrical arc voltage was successfully carried out by acoustical methods [2,3]. The welding arc sound represents the behavior of the electrical parameters of the welding arc, consequently this fact makes possible monitoring the stability of the welding process via sound. The arc sound of the GMA welding in the short circuit transfer mode can represent the extinction and ignition sequence of the arc voltage and therefore it opens the possibility of acoustically detecting perturbations in the welding arc [4]. The main advantage of the sound monitoring system lies in the fact that there is no need to have electrical connections to the welding process since the sound is transmitted from the welding arc to the acoustic sensor through the air. This facilitates the installation of the sensor and reduces the possibility of altering the electric parameters of the welding process and reduces the influence of the electromagnetic field on the acoustic sensor. Some acoustical monitoring systems for GMA welding processes can be found in the literature, but they are not yet used in the industry [5–15]. In the present work a weld defect detection technique based on welding stability evaluation through the sound produced by the welding electric arc was developed.

### 1.1. Welding Electrical Arc and Acoustical Signals

In the present work the relationship between sound pressure, sound pressure level and spectrum frequency profile behavior with the arc voltage and welding current have been studied. The sound pressure is a longitudinal mechanical wave, produced by the difference of pressure in a medium that can be solid, liquid or gaseous; in this work the transport medium is the air. The metallic transference in the welding process produces changes in the air volume on the electric arc environment. This change produces pressure variations that are transported through air and sensed by a microphone. The sound pressure from the electric arc is a consequence of the amplitude modulation of the arc voltage and welding current [2,3]. This relation is expressed by Equation 1.

The sound pressure level—SPL—also called equivalent continuous sound pressure level, is a comparative measurement with the microphone sensitivity. It is defined as twenty times the base ten logarithm iof the ratio of a root-mean—square sound pressure during a time interval to the reference sound pressure. Equation 2 expresses the sensibility function.

(1) $$S_{a}\;(t)=\frac{d(k.V(t).I(t))}{\mathrm{dt}}$$

where, *Sa (t)* is the sound signal (V), *V (t)* the arc voltage (V), *I(t)* welding current (A) and *K* the geometric factor.

(2) $$\mathrm{SPL}=20.\mathrm{Log}\left[\sqrt{\frac{1}{\Delta t}\;\int_{t}^{t+\Delta t}\;P^{2}\;(\xi )d\xi }/p_{o}\right]$$

The relation between the microphone pressure response and its sensibility is give by Equation 3 and therefore the SPL in function of the sound pressure is give by Equation 4. Relating the results of Equations 1 and 4, Equation 5 expresses the SPL in function of the arc voltage and welding current:

(3) $$P(\xi )=\frac{S(\xi )}{50E-3}$$

(4) $$\mathrm{SPL}=20.\mathrm{Log}\left[\sqrt{\frac{1}{\Delta t}\;\int_{t}^{t+\Delta t}\;{\left(\frac{S(\xi )}{50E-3}\right)}^{2}d\xi }/p_{o}\right]$$

(5) $$\mathrm{SPL}=20.\mathrm{Log}\left[20\sqrt{\frac{1}{\Delta t}\;\int_{t}^{t+\Delta t}\;{\left(\frac{d(k*V\;(\xi )*I\;(\xi ))}{d\xi }\right)}^{2}d\xi }/p_{o}\right]$$

In which SPL is the sound pressure level, V the arc voltage, I the arc current, K the geometrical factor, Po the reference sound pressure (20 uPa), ξ is a dummy variable of time integration over the mean time interval, t the start time of the measurement, Δt the averaging time interval, S the sound signal.

For the spectrum frequency profile analysis the continuous Fourier transform has been used; it is a linear transformation that converts the acoustic pressure signal from the time domain to the frequency domain. This transformation is made using the Discrete Fourier Transform - DFT and it is expressed by Equation 6.

(6) $$S\;(k)=\frac{1}{N}\;\sum_{n=0}^{N-1}\;s\;(n)e^{-j2\pi \mathrm{kn}/N}$$

Octave frequency fractions analysis allows one to evaluate the behavior of frequency strips instead of any frequency. A frequency octave is defined as an interval among two frequencies where one of them is the double of the other. The octave band limits are calculated by Equations 7 to 9. After obtaining the acoustic pressure spectra *S(k)*, the octave frequency strips *G(n)* is obtained from Equation 10.

(7) $$f_{C_{n+1}}=2^{m}\;f_{C_{n}}$$

(8) $$f_{L_{n}}=\frac{f_{C_{n}}}{2^{m/2}}$$

(9) $$f_{U_{n}}=2^{m/2}\;f_{C_{n}}$$

(10) $$G(n)=\sqrt{\frac{1}{\left(f_{U_{n}}-f_{L_{n}}\right)}\;\sum_{f(k)=f_{L_{n}}}^{f_{U_{n}}}\;{\left[S(f)\right]}^{2}}$$

where *m* is the octave band fraction, *f_Cn_* central frequency of the *n* band, *f_Ln_* the inferior limit of the *n* band and *f_Un_* the superior limit of the *n* band.

### 1.2. Quality Control in the GMA Welding Process

The study of quality control in the welding processes has been a subject of great interest for many researchers. The task of evaluating weld quality is not trivial, even for the experienced inspector. This is particularly true when it comes to specifying in quantitative terms what attributes of the weld affect its quality and to what extent. Different types of discontinuities have been categorized for this purpose, such as cracks, porosities, undercuts, microfissures, etc. [16]. Generally, good quality GMA welds are uniform and contain little or no artifacts on the bead surface. Furthermore, the bead width is relatively uniform along the length of the bead [17]. To achieve a standard weld quality it is fundamental to maintain continuity of welding stability and this happens when the mass and heat flow of the end of a consumable electrode to the fusion pool through the arc maintains uniformity in the transference; possible discontinuities and/or upheavals in the transference could originate weld disturbances. The stability of the short circuit gas metal arc welding process is directly related to weld pool oscillations [18]. Optimal process stability corresponds to maximum short-circuit rate, minimum standard deviation of the short-circuit rate, a minimum mass transferred per short circuit and minimum spatter loss [19–22]. In the present work, the welding stability was evaluated using the sound pressure through the acoustic ignition frequency (AIF) and sound pressure level (SPL) signatures.

## 2. Measurement and Experimental Method

**Figure 1.**
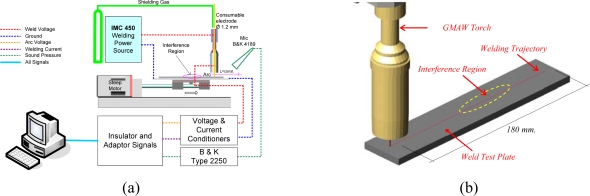
(a) Experimental Setup. (b) Welding Position.

Virtual instrumentation software [23], data acquisition card [24], welding power source [25] and the setup as shown in the Figure 1(a) were used for the acquisition of data based on arc voltage, welding current and arc sound pressure. These parameters were sampled at 20 kHz. The sound pressure was measured using the analogical output decibelimeter [26] which use a microphone 4189 with–26 dB ± 1.5 dB, 50 mV/Pa sensitivity. The microphone was located at approximately 150 mm from the weld pool. The welds were deposited on AISI 1020 (30 mm × 200 × 650 mm) steel plates using 1 mm diameter AWS A5.18 ER70S-6 electrode wire. The shielding gas was a M21 mixture of argon and carbon dioxide (ATAL 5A/Ar 82% + CO_2_ 18%). The induced interferences were located in the center of the welding path, in the so-called weld interference region [Figure 1(b)].

The weld run experiments were carried with the set welding parameters shown in Table 1. The arc voltage varies from 15.0 V to 23.5 V and an arc voltage that generates the weld beads with most stability was chosen experimentally.

In order to have adequate arc voltage identification, four welding sets, as shown in Table 2, were chosen to deposit welds in a flat position following a rectilinear trajectory of 180 mm (Figure 1b). The first set are weld runs without interferences and the others had induced disturbances in the interference region, as shown in Figure 1b. The disturbances for each welding run set are the CTWD variation, grease presence and absence of shielding gas respectively.

## 3. Experimental Results and Discussion

### 3.1. Welding Arc Electrical and Acoustical Signals

**Figure 2.**
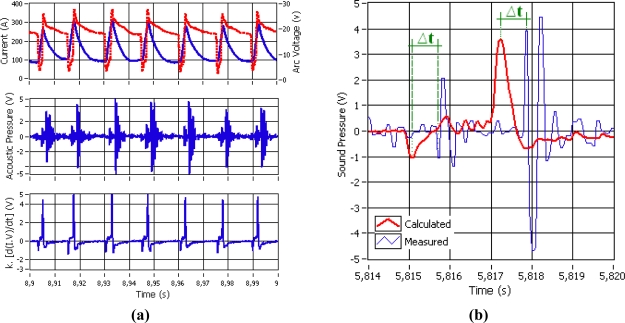
(a) Welding Signals (b) Sound Pressure calculated and measured.

The metallic transference on the short circuit mode in GMA welding (GMAW-S) is characterized by a sequence of ignition and extinction arc cycles. Figure 2a show the behavior of the arc welding current and voltage signals, as well as the welding arc sound and the resultant signal using the Equation 1. The sequence cycles of the welding metallic transference are replicated in the electrical measured signal as well as in the welding arc sound. The arc ignition produces a large acoustical peak and the arc extinction produces a small amplitude acoustical peak. Nevertheless, it is possible to observe a delay ‘Δt’ between the similar signals of the calculated and measured arc sound (see Figure 2b). Some studies in psychoacoustics have determined that as long as the delay of the welding arc sound signal does not exceed 400 ms, the sound will be a good indicator of welding process behavior [27,28]. In our case the Δt delay measured was approximately 0.6 ms, and this value ensures the reliability for monitoring the welding process behavior.

**Figure 3.**
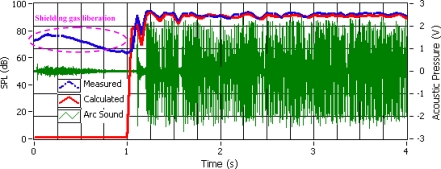
Measured and Calculated SPL.

The welder uses his experience and ability to learn and recognize acoustical signatures from quality welds. Figure 3 shows the measured and calculated SPL (using Equation 5) with its respectively sound pressure signal. It can be observed that the shielding gas flux is sensed by the sound signal, but as the SPL calculated is only function of welding current and arc voltage, this sound signal is not taken into the account. Other information can be obtained from the SPL sound pressure like the arc welding ignition and extinction average frequency, the average period of the transferences cycles and its standard deviations.

**Figure 4.**
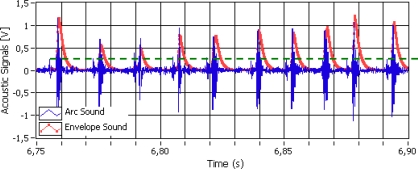
Acoustical Pressure and its envelope Signals.

The measurement of ignition and extinction average frequency of the welding arc is a method for evaluating welding stability [21]. As it was explained before, the arc sound pressure follows the arc ignition and extinction sequence (Figure 2b). The acoustic amplitude pulses produced by the arc ignitions are greater than the acoustic amplitude pulses produced by the extinctions (short circuits). These acoustical impulse sequences occur together with chaotic transients and noise oscillations and in order to reduce it and obtain only the ignition and extinction average frequency, the envelope sound signal was extracted from the acoustic sound signal (see Figure 4).

The envelope sound signal was obtained using a quadratic demodulator. Squaring the signal effectively demodulates the input by using itself as the carrier wave. This means that half the energy of the signal is pushed up to higher frequencies and half is shifted towards DC. The envelope can then be extracted by keeping all the DC low-frequency energy and eliminating the high-frequency energy. However, a “Kalman filter” statistical filter was used due to the fact the sound pressure has a stochastic behavior and when low-pass filters with an elevated order were needed, this order produced a pronounced delay and deformation in the enveloping signal. This statistical filter, instead of letting low frequencies pass, follows the statistical tendency of the squared signal obtained in the enveloping sound pressure [4]. In Figure 4, the 150 ms moving window data was extracted from the sound pressure signal. From the envelope sound pressure signal the arc ignitions for each moving window data are calculated. An ignition takes place whenever the envelope sound pressure signal surpasses the ignition threshold established (k = 0.2).

### 3.2. Weld Bead Quality Profile Identification

**Figure 5.**
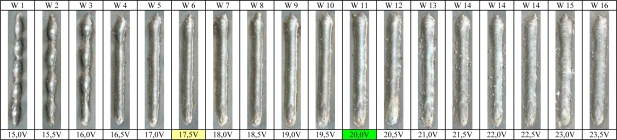
Weld Run Experiments.

Prior to the detection of interferences, many weld experiments for finding the optimal set of the welding parameters were carried out. The satisfactory parameter selection allows reaching the maximum stability in welding, but to reach that it was necessary to choose the adequate arc voltage. Sixteen experiments varying the voltage from 15.0 V to 23.5 V with a 0.5 V step were carried out using the welding parameters shown in Table 1. Figure 5 shows the results of the set of welding experiments.

**Figure 6.**
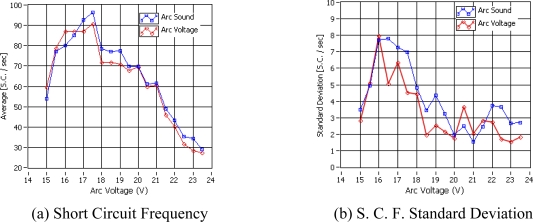
Short Circuits vs Arc Voltage.

Figure 6a shows the relationship between the short circuit frequency calculated by the arc voltage and arc sound and in figure 6b the respective standard deviation is shown. The average short circuit frequency obtained from the arc voltage and the arc sound pressure show a similar result. Moreover, in both the short circuit frequency and standard deviation display some differences between calculation methods, but these differences are minimal and the acoustical method to calculate this statistical parameter can be considered reliable. Related research [21] was followed to choose the arc voltage value that generated the best quality bead (continuity and uniformity of the bead ruggedness) that concluded that the best quality is achieved basically when the short circuit rate is maximum and its standard deviation is minimal, but some unexpected results were discovered. The arc voltage that generates the maximum short circuit rate (17.5 V) is not the weld with the better quality in the weld run experiments (Figure 5). Considering that the arc voltage that generates the minimal standard deviation from the short circuit rate obtained from arc voltage (23.0 V) is not the weld with the better quality and considering that the minimal standard deviation obtained from the arc sound is also not the weld with the better quality, but inferring from the visual inspection of the bead set shows that the weld bead run with the voltage range between 19.0 V and 20.5 V can be considered as the best quality weld bead inside this set.

**Figure 7.**
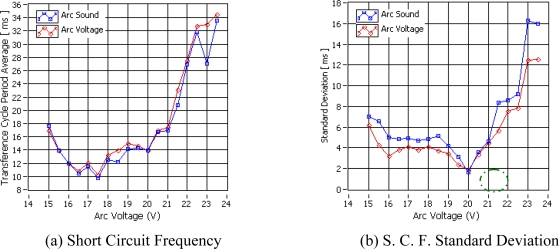
Transference cycle Period vs. Arc Voltage.

In order to chose the arc voltage value that generates the best weld bead quality a second statistical analysis was carried out. Figure 7a, illustrates the relationship between the transference cycle period average obtained by the arc voltage and arc sound at the same different arc voltage values analyzed previously. In this case the results obtained by the electrical and acoustical methods show a narrow similarity with differences of milliseconds. The arc voltage that generates the least transference cycle period average was also 17.5 and comparison with the weld bead quality shows it is not the best. The transference cycle period uniformity is measured by its standard deviation as shown in Figure 7b. This standard deviation distribution has more uniformity than the short circuit frequency standard deviation and the arc voltage that generates the minimal value into standard deviation distribution (20.0 V) also generates the weld bead with more geometrical ruggedness and uniformity and can be considered as the best quality weld bead. Consequently, the remaining weld run experiment for interferences detection were carried out using the welding parameters shown in Table 1 with the arc voltage adjusted to 20.0 V.

### 3.3. Acoustical Profiles to Interferences Detection

**Figure 8.**
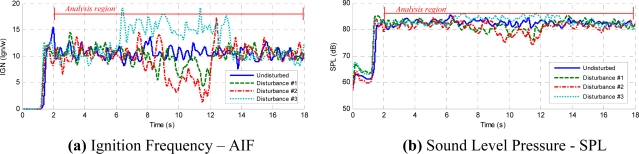
Time Profiles to welding arc sound.

The general approach used to evaluate GMA welds was by examining the variation of weld profiles, sampled across the weld bead in a series of locations. A high quality weld would generally yield a small variation in the weld profiles, while low quality weld profiles would vary substantially, as irregularities and various discontinuities are encountered in the distinct profile scans [17]. The initial weld profiles tested were the arc sound ignition frequency, the average sound pressure level and the power spectral density using the continuous and octave fraction frequency domain. Figure 8a, shows the AIF and 8b, the SPL weld profiles’ signal behavior. These profiles were tested on welding runs with and without the presence of disturbances. Both profile signals were determined using a moving windows signal applied on the arc sound pressure signal. The moving window was fixed at 150 ms considering that the data sample rate was 20 kHz.

**Figure 9.**
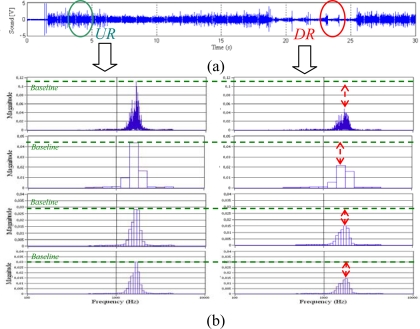
Frequency Profiles to welding arc sound.

Signature analysis of the short-circuiting frequency using the time – frequency analysis method was applied to the welding arc sound. Figure 9a shows the arc sound signal. In this signal there are two regions (undisturbed–UR and disturbed - DR regions). In these regions a spectral analysis was made at continuous and an octave fractions frequency. Figure 9b illustrates the continuous, 1/3, 1/10 and 1/12 octave frequency distributions. When the welding entered the interfered region the spectra frequency varies its magnitude on the overall frequency (see DR in Figure 9b). The spectra frequency magnitude varies on the two analyzed regions; there are approximately dominant frequency components at 2 kHz.

The octave fraction spectra also have greater amplitude inside this band frequency, but it does not show frequency bands signatures that vary pronouncedly with the disturbed sound signals. As there are not signature bands in fraction octave analysis, the continuous frequency versus time behavior was analyzed.

**Figure 10.**
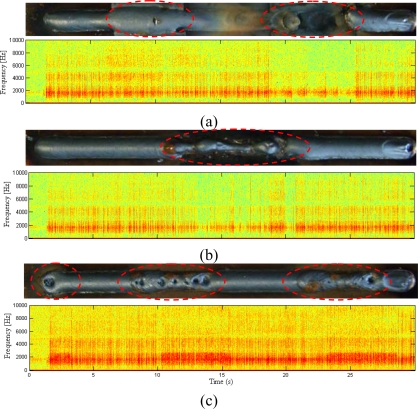
Welding Arc Sound Spectrogram.

Figure 10 shows the spectrogram for welding runs with induced interferences on the plate. The Figures 10a–c correspond to welding experiments with interferences induced by arc length variation, dirty grease on the plate and absence of shielding gas, respectively. In these time-frequency results it is not possible to appreciate any signature frequencies that could identify the different induced interferences. However are possible different amplitude variations for each induced interference. The amplitude variations on the sound spectra imply that there are signature variations on the time domain. The welding arc sound have many chaotic transients between impulses (Figure 9a), these fluctuation have a stochastic nature. Fourier analysis is very effective in problems dealing with frequency location. However, there are severe problems with trying to analyze transient signals using classical Fourier methods [29]. This is the principal reason for not distinguishing clear signatures frequencies on the arc sound spectra that can identify disturbances. The signatures in the time domain described in Figure 8 show pronounced signatures when the welding enters interfered regions.

### 3.4. Interferences Monitoring and Detection

Disturbance detection was made using a limit control based on the third standard deviation method. This control shows optimal results in the disturbances detection of both, electric arc voltage and welding current monitoring signals [16,30]. As already explained, the signature signals analysis was made on a 150 ms moving window. Equations 11–13 represent the average, standard deviation and disturbances control limits for each moving window data respectively.

(11) $$\bar{x_{i}}=\frac{1}{n}\;\sum_{j=1}^{n}\;x_{i}=\frac{1}{n}\left(x_{1}+...+x_{n}\right)$$

(12) $$S_{i}=\sqrt{\frac{1}{n}\;\sum_{j=1}^{n}\;{\left(x_{j}-\bar{x_{i}}\right)}^{2}}$$

(13) $$\mathrm{Control\_Limits}=\bar{P_{N}}\pm 3\times S_{P}$$

where:

$\bar{x_{i}}$ Average parameter of the *i* analysis moving windows (150 ms)
*x_i_* data *i* from the moving window
*n* Data component number from analysis moving window
*S_i_* The standard deviation for the *i* analysis moving window data,
*x_j_* The j component data from analysis moving window data,
$\bar{P_{N}}$ Average established for each parameter,
*S_P_* The standard deviation established for each acoustical parameter without

**Figure 11.**
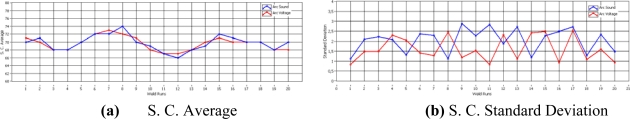
Short circuits average and standard deviation.

In order to validate the disturbances detection method and based on the acoustics of the GMAW-S signal, a total of forty welding runs were carried out (see Table 2). The average short circuit numbers per second obtained from arc voltage and the arc average ignition numbers per seconds obtained from sound pressure show a similar result, as shown in Figure 11a. These results were obtained in the first group of weld experiments without induced disturbances. The minimal standard deviation in Figure 11b confirms that the arc sound pressure can represent well the behavior of the GMAW-S metallic transference.

**Figure 12.**
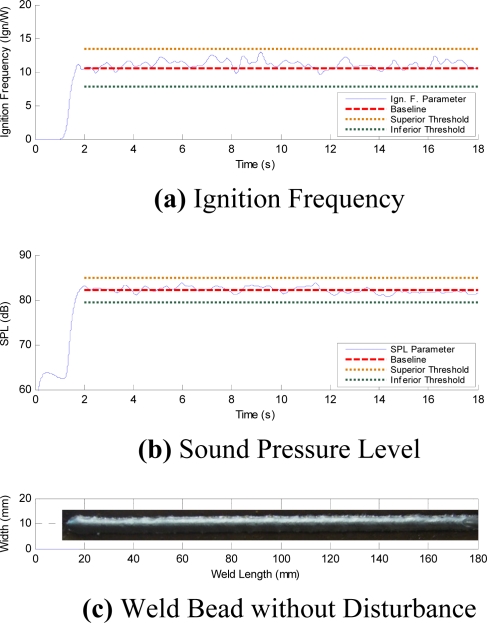
Parameters without Disturbance.

**Figure 13.**
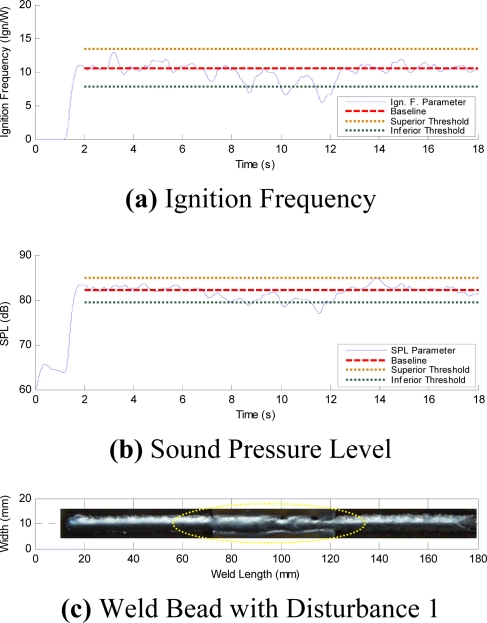
Parameters with Disturbance 1.

**Figure 14.**
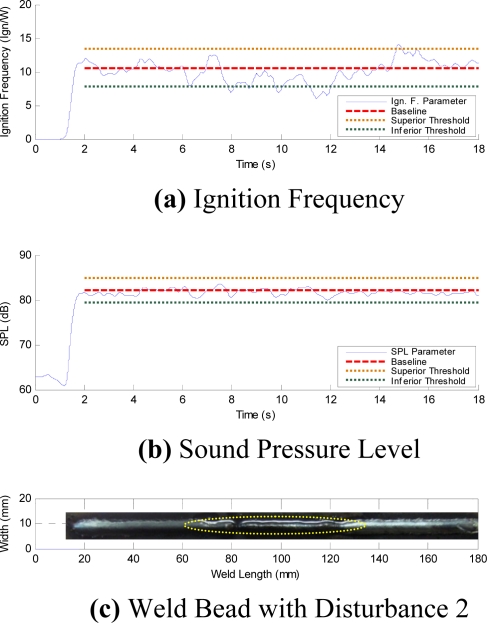
Parameters with Disturbance 2.

**Figure 15.**
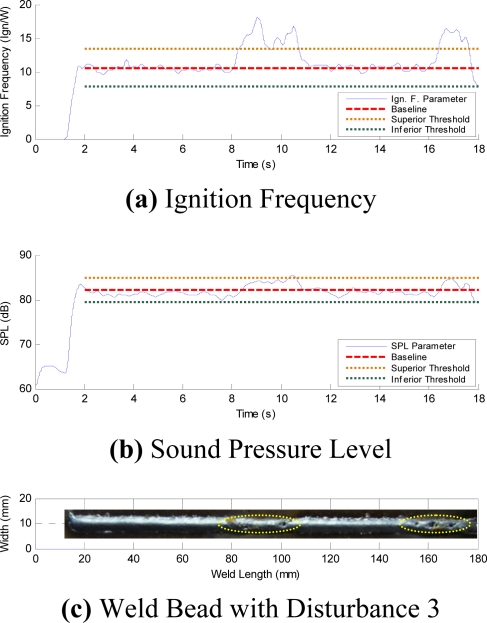
Parameters with Disturbance 3.

The initial time profiles have temporary instabilities (Figures 8a and b). In order to avoid any influence of these initial instabilities on the quality control evaluation, the analysis region is established from second 2 to second 18. Figures 12 to 15a, b and c, respectively, show the acoustical parameters’ behavior and a baseline and two threshold levels, one superior and another inferior, can also be observed. These established limits are three times the standard deviation on the average of each parameter in stable condition welds (without the presence of disturbances). When the parameters are within these two threshold limits are no apparent disturbances in the welds. Therefore, when the parameters exceed the established threshold limits this implies having detected some disturbance that possibly could originate some weld defect. Figures 12 a and b show the AIF and the SPL behaviors, respectively, obtained from the acoustical of arc without induced disturbances. In Figure 12 c the aspect of the welding bead is shown, and even when oscillations on the signal appear, it does not exceed the established threshold limits. These oscillations seem due to the stochastic behavior of the acoustical pressure emitted by the electric arc of the welding process and do not necessarily represent the presence of disturbances.

Figures 13a and b show the AIF and the average SPL per moving window respectively. Both graphics were obtained from the acoustics of a welding arc with induced disturbance originated by the variation of the arc length. Figure 13c shows the visual aspect of the weld bead. The instabilities only occur when the weld bead passes through the beginning, end and the holes of the added plate, respectively. In Figures 13a and b it can also be observed that the ignitions frequency and the SPL do not present oscillations that exceed the established threshold limits, before and after the weld bead passes throughout the interference region. When the weld passes throughout the interference region, abrupt changes of signal level are produced in each parameter. These instabilities exceed the level control previously established. When the CTWD length varies, the arc length varies too, these variations produce instabilities in the arc ignition. It can also be observed that the parameters return to inside threshold limits even without leaving the disturbance region due to the fact the arc reaches a new level of stability.

In Figures 14 a, b the acoustical stability parameters behavior for a weld bead with a disturbance induced due to grease presence in the welding trajectory is shown (see Figure 14c). When the welding run passes the disturbance region, instabilities in the arc ignitions were observed and unexpected upheavals in the metallic transference cycles occurs forming weld bead deposit interruptions in all welding trajectories. Initially, when the weld run reaches the grease no oscillations take place in the ignition frequency due to the evaporation of the grease borders caused by the thermal welding cycles. In the AIF parameter it can be observed that the interference is noticeable as a chaotic decrease, however this behavior overcame slightly the control limits (see Figure 14a).

The average SPL parameter has little abnormal variations when the weld run passes the interference. The induced interference generates structural discontinuities in the weld bead but only in AIF parameter this is is most evident than the SPL parameter. Figures 15a and b show the AIF and SPL parameter behavior in welding experiments without shield gas. In this case the induced perturbations were localized in two regions on the weld bead. These interferences have lead to formation of porosities (see Figure 15c) and higher spatter level. The AIF parameter has increased suddenly and this behavior is shown in both instabilities and it exceeds the established control limit. The AIF were increased because the absence of the shield gas causes contamination in the arc welding environment causing incomplete metallic transference, increasing the short circuit and ignitions rate. This increment, noticeable in the AIF, is also noticeable in the SPL, nevertheless these variations do not exceed the threshold limit control.

## 5. Conclusions

Arc voltage and sound pressure signals were tested for monitoring the welding process. As both signal have the same behavior, it can be concluded that the sound pressure can be used for welding monitoring. From the sound pressure two parameters were calculated: AIF and SPL. The monitoring of the GMAW-S process by digital analysis of acoustical welding parameters enables one to detect disturbances related to phenomena that take place in the welding arc and can influence its stability. This fact shows that the arc acoustic is a potential non-intrusive tool that could be used for weld quality evaluation.

The induced interference CTWD variation affects the length of wire extension and the presence of grease together with the absence of gas shielding affect the whole protection of the welding pool. In the first case the defects caused by this type of disturbances can be unrecognized by the average SPL parameter but the AIF is most expressive and overcomes the control limits. In the second case the presence of grease and the lack of shielding gas affect the whole shielding welding arc. Oxygen, nitrogen and hydrogen as components of the air or the products of grease decomposition change the welding arc shield properties such as ionization potential, the surface tension of molten metal, the condition of welding arc ignition and other essential conditions of arc burning. These interferences have been easily detected by the AIF parameter but the SPL, even though it presents abnormal variations, it is not exceeds the control limits. For all welding experiments, the SPL did not have an expressive response to the presence of perturbations in comparison with the AIF parameter that could be noticed in the three sets of weld run experiments.

The instability profiles make possible the identification of each type of induced disturbance. The instabilities and disturbance identification in real time based on the acoustics of the electric arc could become a good control tool for the GMA welding process.

## Figures and Tables

**Table 1.** Welding Parameters Set.

**Voltage [V]**	**Wire Speed [m/min]**	**Speed Welding [mm/s]**	**CTWD [mm]**	**Shield Gas flow [L/min]**
15.0 – 23.5	6	10	12	15

**Table 2.** Welding Experimental Set.

**Weld Set**		**Number of Runs**	**Induced Disturbance**
1	Without Disturbances	10	Without Disturbances
2	CTWD variation	10	CTWD variation (from 12 to 10 mm)
3	Greasy Presence	10	Presence of Grease
4	Shield Gas Absence	10	Absence of Shield Gas
